# The Emergence of the Spike Furin Cleavage Site in SARS-CoV-2

**DOI:** 10.1093/molbev/msab327

**Published:** 2021-11-12

**Authors:** Yujia Alina Chan, Shing Hei Zhan

**Affiliations:** 1 Stanley Center for Psychiatric Research, Broad Institute of MIT and Harvard, Cambridge, MA, USA; 2 Department of Zoology & Biodiversity Research Centre, University of British Columbia, Vancouver, BC, Canada

**Keywords:** COVID-19, furin cleavage site, virology, coronavirus

## Abstract

Compared with other SARS-related coronaviruses (SARSr-CoVs), SARS-CoV-2 possesses a unique furin cleavage site (FCS) in its spike. This has stimulated discussion pertaining to the origin of SARS-CoV-2 because the FCS has been observed to be under strong selective pressure in humans and confers the enhanced ability to infect some cell types and induce cell–cell fusion. Furthermore, scientists have demonstrated interest in studying novel cleavage sites by introducing them into SARSr-CoVs. We review what is known about the SARS-CoV-2 FCS in the context of its pathogenesis, origin, and how future wildlife coronavirus sampling may alter the interpretation of existing data.

Coronaviruses (CoVs) are named for the crown-like spike surface glycoproteins on the viral envelope. Spikes consist of two domains: S1 for receptor binding and S2 for membrane fusion. Upon encountering a host cell, S1 binds to a receptor protein on the cell surface, and then S2 mediates membrane fusion for viral entry into the cell. Spikes are primed for fusion activation by proteolytic cleavage, which can occur at the S1/S2 boundary (typically during spike protein synthesis) and at the S2’ adjacent to the S2 fusion peptide (Whittaker et al. 2021). Whether S1/S2 cleavage in the spikes of all animal CoVs is obligate remains to be further investigated. S1/S2 cleavage has been found to be efficient in some CoVs such as MERS-CoV and infectious bronchitis virus (IBV) while undetectable in others depending on the host cell system and the presence of different proteases (Belouzard et al. 2009; Li et al. 2015). For example, in Severe Acute Respiratory Syndrome (SARS)-CoV, S1/S2 cleavage is not observed in COS-7 or CHO cells but weak cleavage can be seen in CHO cells over-expressing furin or in BHK cells (Follis et al. 2006; Belouzard et al. 2009); S1/S2 cleavage in the SARS-CoV spike can only be achieved by a limited repertoire of proteases and to a lower efficiency compared with the SARS-CoV-2 spike (Jaimes et al. 2020).

This cleavage-mediated activation is complex and often involves multiple host proteases (Hulswit et al. 2016). For a detailed review of spike activation mechanisms, please see Whittaker et al. (2021). Cleavage by proprotein convertases, such as trypsin- or furin-like enzymes, can trigger conformational changes that release the S2 fusion peptide for insertion into the cell membrane, which initiates membrane fusion and viral entry. After infecting a cell, expression and localization of the spike to the host cell membrane can induce cell–cell fusion among neighboring cells (forming multinucleate enlarged cells called syncytia), which has been hypothesized to facilitate virus spread among fused cells without exposure to neutralizing antibodies (Fehr and Perlman 2015). For example, in COVID-19 patients, the surface epithelial cells of the alveoli often form syncytia, which has been suggested to be due to the ability of the SARS-CoV-2 spike to be primed by furin at the plasma membrane, similar to MERS-CoV (Bussani et al. 2020; Xu et al. 2020).

In some CoVs, the spike protein is effectively processed at furin cleavage sites (FCS; minimal motif of R-X-X-R) that can occur at the S1/S2 boundary, which plays a key role in the cell tropism and pathogenesis of these viruses (Yamada and Liu 2009; Millet and Whittaker 2014; Coutard et al. 2020). For example, IBV and some feline and canine CoVs possess a highly polybasic R-R-X-R-R motif in their spikes; these can be lost during passaging in cell culture, for example, RRSRR to RRSRG, potentially in exchange for the increased ability to utilize heparan sulfate or other novel entry receptors (de Haan et al. 2008). Strikingly, in human coronavirus OC43, one of the common cold CoVs, more recent isolates that had not been passaged in cell culture were found to possess an RRSRR motif in the spike as compared with the RRSRG observed in passaged OC43 strains (Vijgen et al. 2005; de Haan et al. 2008). In SARS-CoV-2, structural studies have suggested that furin cleavage at the S1/S2 boundary primes the spike for an open conformation required for binding to the ACE2 entry receptor (Wrobel et al. 2020).

Similar proteolytic cleavage has been characterized in all class I viral fusion proteins, such as those of human immunodeficiency virus, influenza virus, Ebola virus, and respiratory syncytial virus (Hallenberger et al. 1997; Basak et al. 2001; Whittaker 2021). For a detailed review of class I and II viral fusion proteins, please see Rey and Lok (2018). Scientists have thus been interested in understanding how the virus’ ability to infect different cell types and animal models might be affected if cleavage sites were introduced.

Focusing on CoVs, several groups have shown that introducing a cleavage site to enable promiscuous cleavage of the spike by different proteases can boost viral entry in different cell types. For instance, in 2015, scientists who introduced an FCS into the spike of a porcine epidemic diarrhea CoV found that the resulting virus exhibited trypsin-independent cell–cell fusion and an increased ability to infect different cell types (Li et al. 2015). That same year, another group of scientists manipulated the S1/S2 FCS and a second cleavage site in the spikes of MERS-CoV and a MERS-related virus to show that these could enable the MERS-related CoV spike to become activated by human proteases and mediate viral entry into human cells (Yang et al. 2015). Scientists have also been introducing FCSs into the spike of SARS-CoV to gain a better understanding of how an additional FCS can alter membrane fusion and reliance on cellular proteases (Follis et al. 2006; Belouzard et al. 2009).

Against this backdrop of scientists introducing FCSs into the spikes of various CoVs including SARSr-CoVs, the discovery of a unique FCS at the spike S1/S2 boundary in SARS-CoV-2 continues to fuel heated debates about the origin of the virus.

## The Discovery and Characterization of the Unique S1/S2 FCS in SARS-CoV-2

In comparison to all known SARSr-CoVs, SARS-CoV-2 possesses a unique four-residue P-R-R-A (681–684) insertion at its spike S1/S2 junction, producing an FCS. Although the SARS-CoV-2 FCS (P-R-R-A-R) may sometimes be described as “non-canonical” (it is not an R-R-X-R-R), it is highly functional and similar to FCSs found in other CoVs such as MERS (P-R-S-V-R, which is one R short compared with that of SARS-CoV-2).

The SARS-CoV-2 S1/S2 FCS was identified in January and early February by Li et al. (2020) and Coutard et al. (2020) respectively. Li et al. (2020) claimed to be the first to report the FCS on January 21, 2020, and postulated that the “cleavage site may increase the efficiency of virus infection into cells, making 2019-nCoV has significantly stronger transmissibility than SARS coronavirus”. Coutard et al. (2020) suggested that the novel FCS could have “significant functional implications for virus entry”. Another group, Walls et al. observed in their pseudovirion production that, although the SARS-CoV spike remained largely uncleaved at the S1/S2 junction, the SARS-CoV-2 spike was found to have near-complete S1/S2 cleavage; they similarly hypothesized that the FCS could “expand its tropism and/or enhance its transmissibility, compared with SARS-CoV and SARSr-CoV isolates, due to the near-ubiquitous distribution of furin-like proteases and their reported effects on other viruses” (Walls et al. 2020). It was a straightforward deduction for independent groups of scientists that an S1/S2 FCS could confer functional advantages to a SARSr-CoV.

Since then, numerous groups have characterized SARS-CoV-2 FCS deletion mutants in various cell lines and animal models and found that FCS deletion significantly attenuated viral infection; however, the FCS is not required for and can even reduce the rate of SARS-CoV-2 replication in Vero E6 cells (Lau et al. 2020; Johnson et al. 2021; Peacock et al. 2021). Early studies have demonstrated that the FCS confers on SARS-CoV-2 an expanded proteolytic activation profile that can be facilitated by a wide variety of proteases (Jaimes et al. 2020), alongside the enhanced ability to efficiently enter and replicate in Calu-3 (human respiratory) cells and to induce efficient cell–cell fusion (Hoffmann et al. 2020; Papa et al. 2021). In Vero or 293 T cells, SARS-CoV-2 spike expression could induce syncytium formation, which was increased in the presence of proteases, such as trypsin and TMPRSS2; however, there was dramatically less syncytium formation when the S1/S2 site was replaced by that of SARS-CoV, and no detectable syncytium formation even in the presence of proteases when the motif was removed entirely (Hoffmann et al. 2020; Papa et al. 2021). When the *FURIN* gene was knocked out in Vero or 293 T cells, cell–cell fusion was significantly decreased although syncytia were still observed (Papa et al. 2021).

In animal models, Lau et al. (2020) found that, compared with the wild-type SARS-CoV-2, an FCS deletion mutant replicated less efficiently in tracheal and lung tissues, and caused less weight loss and reduced lung damage in the infected hamsters. In corroboration, Johnson et al. (2021) reported that an FCS deletion mutant (ΔPRRA) was less efficient (∼10-fold reduction) than the wild-type SARS-CoV-2 at replicating in a human respiratory Calu-3 2B4 cell line, and also resulted in less weight loss and less severe disease in hamsters and K18-hACE2 transgenic mouse models. Johnson et al. (2021) claimed that their results “demonstrate a critical role for the furin cleavage site in infection with SARS-CoV-2”. In ferrets infected with SARS-CoV-2 lacking the FCS, virus was shed at lower titers and not transmitted to cohoused ferrets in contrast to similar infection and transmission experiments with the wild type SARS-CoV-2 (Peacock et al. 2021).

In humans, mutations impacting the FCS have been rare among the 3,763,000+ high-quality SARS-CoV-2 genome sequences deposited on the GISAID database (accessed October 24, 2021); less than 0.05% of the sequences have mutations at R682 or R683, and even fewer (0.0007%) have mutations at R685 (Elbe and Buckland-Merrett 2017; Shu and McCauley 2017; Chen et al. 2021). Similar observations made earlier in the pandemic have led scientists to suggest that the FCS may be experiencing strong purifying selection in humans (Lau et al. 2020) or other mammals (Liu et al. 2020), albeit the non-Arginine residues in the PRRAR motif continue to be permissive to potential optimization during human viral evolution. For instance, among variants of concern and interest, the Delta and Kappa variants have a P681R mutation, and the Alpha and Mu variants have a P681H mutation. Recently, it was found that the P681R and P681H mutations do not enhance spike protein expression or furin cleavage in HEK293 cells, although the Delta (but not the Kappa) variant spike enabled increased infection of host cells that only express low levels of ACE2 (Zhang et al. 2021).

## How Does the SARS-CoV-2 Spike Compare with Those of Other SARSr-CoVs?

**Fig. 1.**
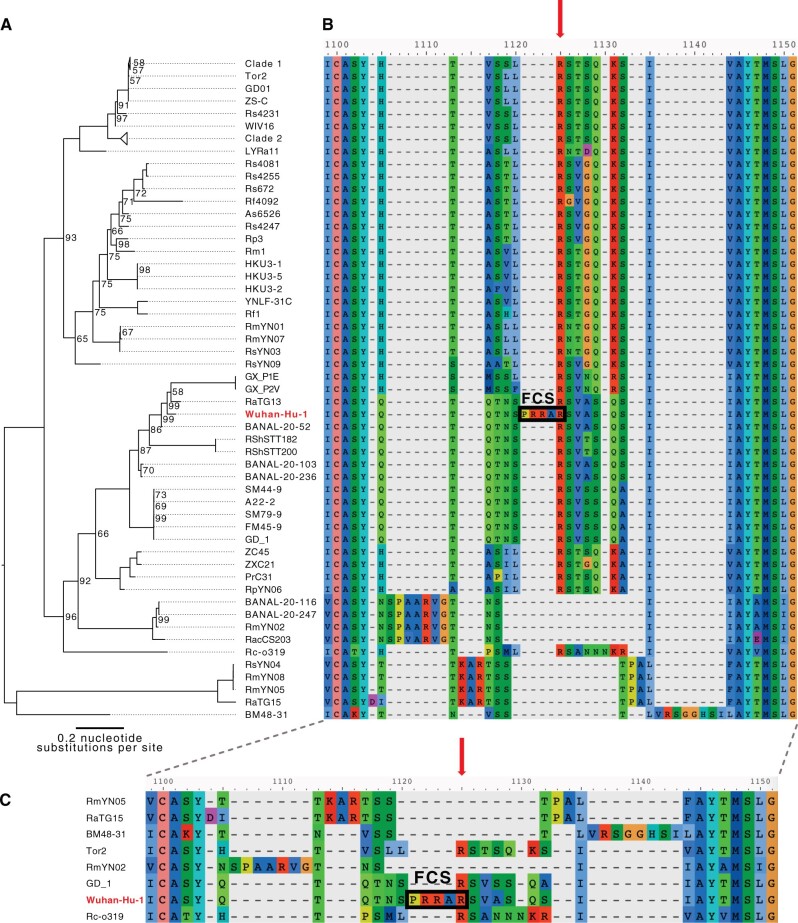
Phylogenetic tree of the spike gene (*A*) and alignments of the S1/S2 region of the FCS by codon sequences (*B*). A codon alignment of the spike sequences was generated using PRANK version 170427 (Löytynoja 2021) and was then translated into amino acid residues for visualization. A phylogenetic tree was estimated from the codon alignment using IQTree version 1.6.12 with the options “-bb 1000 -alrt 1000” (Minh et al. 2020). The consensus tree from IQTree was rooted at midpoint and visualized using FigTree version 1.4.4 (http://tree.bio.ed.ac.uk/software/figtree/). Ultra-bootstrap values less than 100 are labeled in the tree. In Clade 1 and Clade 2, we collapsed the entries, which contained identical amino acid sequences in the 1,100–1,150 region of the full amino acid alignment, and arbitrarily selected SZ3 and WIV1 as the representatives of Clade 1 and Clade 2, respectively. (*C*) To focus on the entries that led to the indels in the S1/S2 region in the full alignment, we collapsed similar entries and subsequently used this visualization to facilitate the comparison of alignments in figure 2. The alignments were visualized using AliView version 1.26 (Larsson 2014). The last arginine (R) residue in the PRRAR motif from Wuhan-Hu-1 is indicated by the red arrows above each alignment. The FCS motif is also indicated by a box and label.

**Fig. 2.**
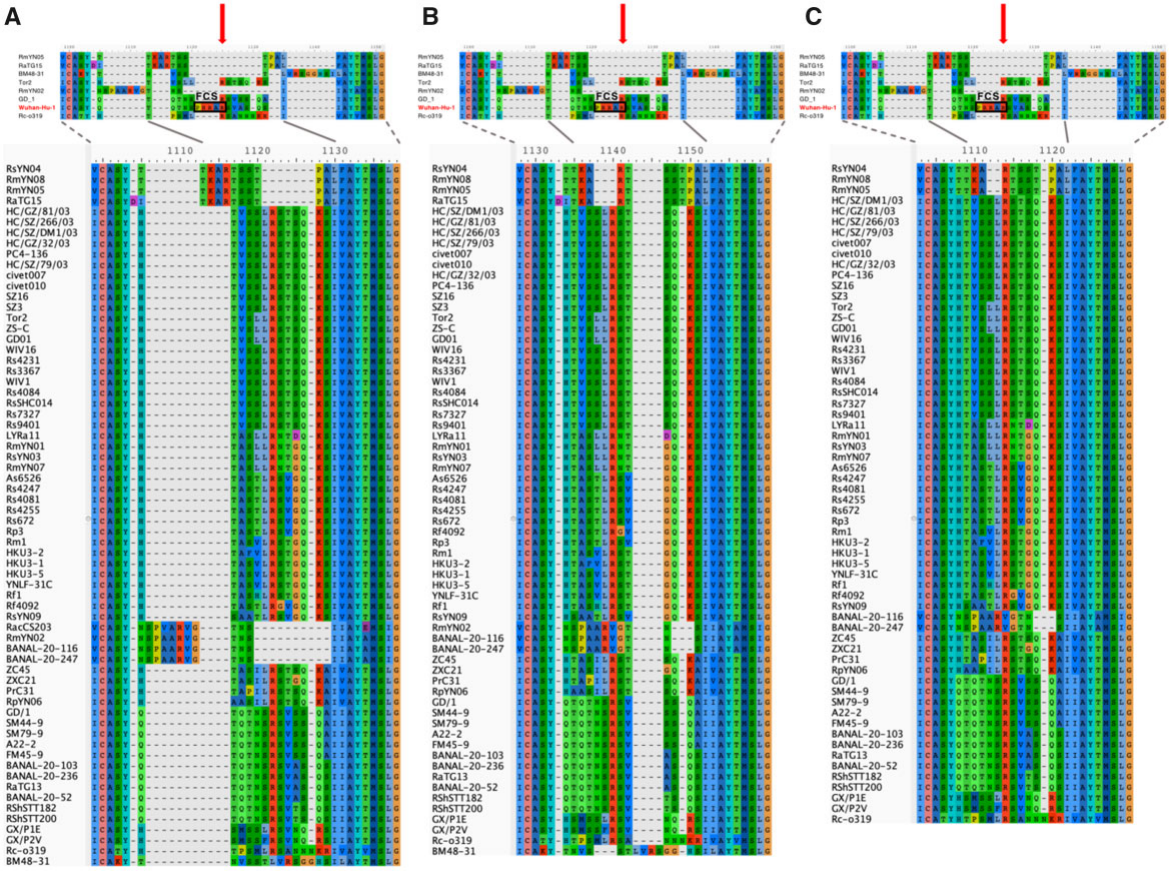
Amino acid alignments of subsets of SARSr-CoV genomes. The subsets were created by excluding select entries to illustrate how sensitive alignments in the spike S1/S2 region can be to lineage sampling. We examined three subsets: (*A*) without Wuhan-Hu-1, (*B*) without Wuhan-Hu-1 and RacCS203, and (*C*) without Wuhan-Hu-1, RacCS203, RaTG15, BM48-31, and RmYN02. The codon sequences from the subsets were aligned using PRANK, translated into amino acid, and then visualized using AliView. A visual comparison between each of the subsets and the full sequence alignment (at the top of each panel; taken from fig. 1*C*) shows that the alignment in the S1/S2 region is sensitive to including one or a few samples with a different amino acid sequence in the region.

Several novel SARS-CoV-2-like genomes from horseshoe bats in Japan (Rc-o319), Cambodia (RshSTT182 and RshSTT200), Thailand (RacCS203, 224, 253, 263, and 271), Laos (BANAL-52, -103, -116, -236, and -247), and China (RpYN03-09) have been published (Murakami et al. 2020; Delaune et al. 2021; Temmam et al. 2021; Wacharapluesadee et al. 2021; Zhou et al. 2021). However, to this day, no known sarbecovirus except SARS-CoV-2 has an FCS insertion at the S1/S2 junction. To understand how unique the S1/S2 FCS in SARS-CoV-2 is, we re-aligned the S1/S2 regions of known SARS-CoV-2-like CoVs. We found that the alignment is sensitive to lineage sampling by visually comparing a codon sequence alignment of all known spikes (fig. 1; supplementary fig. S1, Supplementary Material online) to alignments of a few subsets of the spikes (fig. 2). The full alignment appears to feature numerous insertions at different positions in the S1/S2 region across the range of spike sequences examined (fig. 1). Thus far, the PRRA motif is unique in its position, whereas spike sequences more distantly related to that of SARS-CoV-2 present other commonly featured insertions at other positions. For instance, despite being collected from different locations RmYN02 (Yunnan, China), RacCS203 (Thailand), BANAL-20-116 and BANAL-20-247 (Laos), the NSPXARVG motif appears to be conserved across these four more closely related spikes. It is currently difficult to know exactly how unique the SARS-CoV-2 PRRA motif is among SARS-CoV-2-like spike sequences because these viral lineages are relatively under-sampled.

Based on our analysis, any additional SARS-CoV-2-like spike sequence has the potential to re-inform and shift the alignment over this region and thus dramatically impact interpretations about their evolutionary history. To demonstrate this effect, we examined three alignments of the S1/S2 region created by excluding sequences that introduced indels in the full alignment (fig. 2). Removing SARS-CoV-2 alone was sufficient to change the predicted insertion positions among the remaining SARSr-CoVs (fig. 2*A*). Interestingly, further excluding RacCS203 but not the closely related RmYN02, BANAL-20-116, and BANAL-20-247 (fig. 2*B*) resulted in another dramatic change in the alignment. Upon additionally omitting RaTG15, BM48-31, and RmYN02, the alignment became even more concise (fig. 2*C*). In other words, our analysis showed that a single, new SARS-CoV-2-like spike sequence, from a lineage that has not yet been sampled, has the potential to reshape the S1/S2 region alignment.

Without broader geographical sampling of diverse bat species, we are left with an incomplete and probably biased picture of the evolutionary history of the spikes of SARS-CoV-2-like viruses. Some papers have proposed alignments that suggest insertions at the S1/S2 region in RmYN02 and RacCS203 (Zhou et al. 2020, 2021); however, these still do not result in a polybasic motif or an FCS. From our analysis, these alignments represent only one of numerous possible alignments. In our alignment, these “insertions” are located in a different part of the S1/S2 region (fig. 1; supplementary fig. S1, Supplementary Material online). Regardless, the alignment over the S1/S2 region is prone to revision when new sequences are discovered in the future, especially given that sampling of SARSr-CoVs thus far is poor (fig. 2; supplementary fig. S2, Supplementary Material online). In particular, the S1/S2 region of interest lies in a flexible, solvent-accessible loop (Lemmin et al. 2020), making it challenging to support alignments with protein structure information or to determine the most probable evolutionary history of this region. To complicate the analysis, some of the novel genomes, such as that of RmYN02, exhibit a divergent haplotype that has been suggested to have arisen from recombination events with distantly related viral strains (Wang et al. 2020). Based on the data available at the time of this writing, the S1/S2 region in SARS-CoV-2-like viruses appears to be unconserved and prone to substitutions, indels, and possibly recombinations.

The most closely related spike genes from bat CoVs BANAL-20-52 and RaTG13 (95% and 93% spike nucleotide sequence identity with SARS-CoV-2, respectively), RshSTT182 and RshSTT200, the Guangdong and Guangxi pangolin CoVs, and even the Zhoushan ZXC21 and ZC45 SARS-CoVs do not have an apparent S1/S2 FCS (fig. 1). These observations suggest that among sarbecoviruses, an S1/S2 FCS recently emerged in SARS-CoV-2. Insights from the structure of the SARS-CoV-2 spike also support this hypothesis: the FCS appears to destabilize the spike and the first known adaptive mutation in spike (D614G) is likely to have evolved to primarily compensate for this destabilization (Zhang et al. 2020; Choe and Farzan 2021). Specifically, Zhang et al. (2020) point out that differences between the SARS-CoV-2 spike variants with D614 versus G614 can only be observed when the FCS is functional and actively utilized by the host cell. The alternative hypothesis where the FCS was lost from several other viruses in the same clade, including those sharing very high spike gene identity with SARS-CoV-2, is less parsimonious, especially in consideration of the enhanced functionalities conferred by the FCS on SARS-CoV-2. For this reason, hypothesis generation has generally focused on mechanisms by which the S1/S2 FCS could have emerged in SARS-CoV-2 (Gallaher 2020; Lytras 2020). Nonetheless, due to how under-sampled these viruses are across different host species, it is not straightforward to predict how much recombination and template switching might have occurred to result in SARS-CoV-2. There is an instance where an S1/S2 FCS was lost in a singular CoV, while its close relatives with similar spikes (up to 96% spike gene identity) retained the FCS (Wu and Zhao 2020). However, this is the reverse scenario to that of SARS-CoV-2, which appears to have gained a highly advantageous FCS compared with the rest of its clade.

## The Difficulty of Determining the Origin of the SARS-CoV-2 FCS

Jack Nunberg, whose group first inserted an S1/S2 FCS into the spike of SARS-CoV, said: “there is no way to know whether humans or nature inserted the site” in SARS-CoV-2 (Cyranoski 2020). In 2006, Nunberg’s group had investigated whether the fusion activity of SARS-CoV could benefit from proteolytic cleavage, similar to other CoVs that had been characterized at the time. They inserted a synthetic furin recognition sequence at the putative R667 S1/S2 cleavage site, and demonstrated that an FCS insertion (as compared with a substitution) upstream of R667 markedly increased the ability of the spike to induce cell–cell fusion (Follis et al. 2006). A group led by Gary Whittaker later showed that a substitution was sufficient when combined with the introduction of another FCS at the S2 region; this experiment in SARS-CoV was inspired by the observation of a similar setup in IBV strain Beaudette, which has efficient cleavage at both S1/S2 and S2’ (Belouzard et al. 2009). Whittaker recently (August 2021) published a comment describing the SARS-CoV-2 FCS as “highly unusual” (Whittaker 2021). Similar studies on MERS-CoV had also determined that its S1/S2 FCS is required for efficient entry into human lung and intestine cells, and influences the cell tropism of the virus (Park et al. 2016; Kleine-Weber et al. 2018). In September 2021, it was reported that an international group of scientists (including from the Wuhan Institute of Virology) had, in March 2018, proposed a roadmap for detecting novel proteolytic cleavage sites (including FCSs) in the spike sequences of novel sarbecoviruses and inserting these novel cleavage sites into the appropriate parental strain (Daszak 2018; Lerner and Hibbett 2021). These research endeavors and others described in our introduction relating to the S1/S2 FCS in the context of various CoV spikes have led to speculation that the SARS-CoV-2 FCS could have been similarly inserted to characterize its function in different cell types. The virologist David Baltimore commented that “these features make a powerful challenge to the idea of a natural origin for SARS2,” later clarifying that “you can't distinguish between the two origins from just looking at the sequence” (Caltech Weekly 2021).

There has been intense discussion about exactly how an FCS would be experimentally inserted into SARSr-CoVs. For instance, some scientists have pointed out that the SARS-CoV-2 FCS insertion is out of frame; other scientists have pointed out that the P-R-R-A insertion utilizes a CGG-CGG doublet that might suggest codon optimization (Holmes et al. 2021; Segreto and Deigin 2021). The CGG codon only appears with ∼5% frequency in SARS-CoV-2-like viruses. Hence, some scientists have postulated that a CGG-CGG doublet is improbable in natural SARSr-CoVs. However, the rarity of a codon cannot be used to confidently determine the provenance of, or rule out a possible provenance of, a given doublet. For instance, a similar P-R-R-A-R motif in the spike of a feline CoV has been observed to be encoded with a CGG-CGA for the double Arginine (Bank-Wolf et al. 2014). In this case, a single mutation could change the CGG-CGA to a CGG-CGG. Our position is that, without access to the full set of viral sequences available to scientists prior to the emergence of SARS-CoV-2, it is difficult to know what range of novel cleavage sites might have been experimentally characterized and how these sites might have been introduced into other SARSr-CoVs.

As more bat CoVs are sampled, it is possible that another SARSr-CoV will be discovered with an S1/S2 FCS insertion. FCSs have evolved naturally in other non-sarbecovirus families of betacoronaviruses (Wu and Zhao 2020). Therefore, an S1/S2 FCS emerging in a sarbecovirus is consistent with natural evolution. Even so, the knowledge that scientists had a workflow for identifying novel cleavage sites in diverse SARSr-CoVs and experimentally characterizing these cleavage sites in SARSr-CoVs—likely in a manner that makes the resulting recombinant SARSr-CoV practically indistinguishable from a rare SARSr-CoV with a naturally emerging FCS—makes it challenging to rule out an artificial origin of the SARS-CoV-2 S1/S2 FCS (Daszak 2018; Lerner and Hibbett 2021).

## Supplementary Material

Supplementary data are available at *Molecular Biology and Evolution* online.

## Supplementary Material

msab327_Supplementary_Data
