# Supplementary material for: The Emergence of the Spike Furin Cleavage Site in SARS-CoV-2
Source: Mol Biol Evol. 2021 Nov 12;39(1):msab327. doi: 10.1093/molbev/msab327 (PMC8689951; doi:10.1093/molbev/msab327)
Supplement: msab327_Supplementary_Data [file msab327_supplementary_data.zip › Supplementary Figure legends.pdf]

Supplementary Figure legends

Supplementary Figure 1. Phylogenetic tree of the spike gene (**A**) and alignment of the S1/S2 region of the furin cleavage site (FCS) by codon sequences (**B**). A codon alignment of the spike sequences was generated using PRANK. A phylogenetic tree was estimated from the codon alignment using IQTree with the options "-bb 1000 -alrt 1000" (Minh et al. 2020). The consensus tree from IQTree was rooted at midpoint and visualized using FigTree. Ultra-bootstrap values less than 100 are labelled in the tree. In Clade 1 and Clade 2, we collapsed the entries, which contained identical amino acid sequences in the 1,100-1,150 region of the full amino acid alignment, and arbitrarily took SZ3 and WIV1 as the representatives of Clade 1 and Clade 2, respectively. (**C**) To focus on the entries that led to the indels in the S1/S2 region in the full alignment, we collapsed similar entries and subsequently used this visualization to facilitate the comparison of alignments in **Supplementary Figure 2**. The alignments were visualized using AliView. The last arginine (R) residue in the PRRAR motif from Wuhan-Hu-1 is indicated by red arrows above each alignment. The FCS motif is also indicated by a box and label. Note that the phylogenetic tree and alignment are the same as those presented in **Figure 1**, which shows the alignment in amino acids instead.

Supplementary Figure 2. Nucleotide alignments of subsets of SARSr-CoV spike sequences. The subsets were created by excluding select entries to illustrate how sensitive alignments in the spike S1/S2 region can be to lineage sampling. We examined three subsets: **(A)** without Wuhan-Hu-1, **(B)** without Wuhan-Hu-1 and RacCS203, and **(C)** without Wuhan-Hu-1, RacCS203, RaTG15, BM48-31, and RmYN02. The codon sequences from the subsets were aligned using PRANK, translated into amino acid, and then visualized using AliView. A visual comparison between each of the subsets and the full sequence alignment (at the top of each panel; taken from **Supplementary Figure 1C**) shows that the alignment in the S1/S2 region is sensitive to including one or a few samples with a different amino acid sequence in the region. The last arginine (R) residue in the PRRAR motif from Wuhan-Hu-1 is indicated by red arrows above each alignment. The FCS motif is also indicated by a box and label. Note that the phylogenetic tree and alignment are the same as those presented in **Figure 2**, which shows the alignment in amino acids instead.
