# Supplementary figures and images for: The Emergence of the Spike Furin Cleavage Site in SARS-CoV-2
Source: Mol Biol Evol. 2021 Nov 12;39(1):msab327. doi: 10.1093/molbev/msab327 (PMC8689951; doi:10.1093/molbev/msab327)

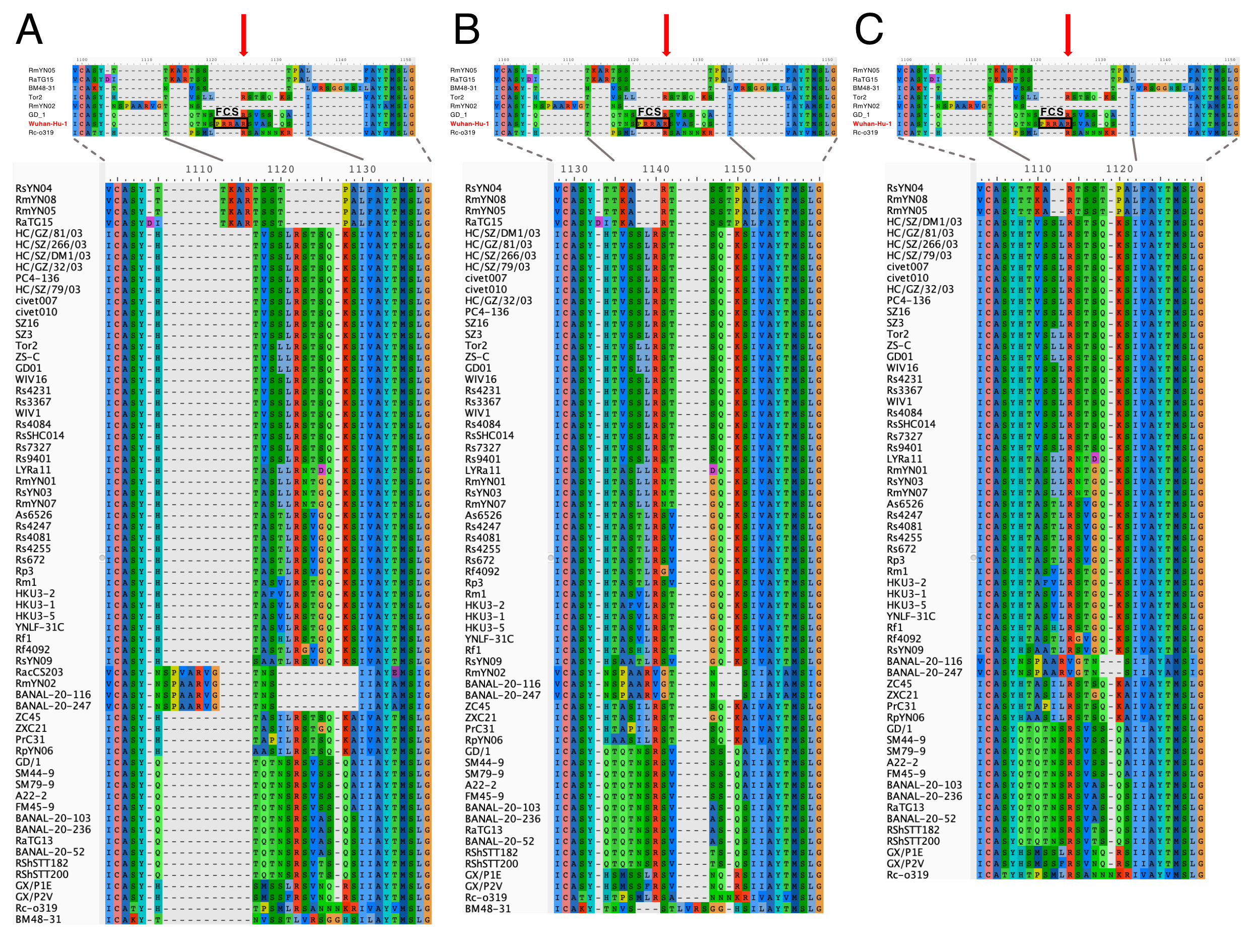

Supplement: msab327_Supplementary_Data [file msab327_supplementary_data.zip › Figure2_ChanZhan_Nov8.png]

A

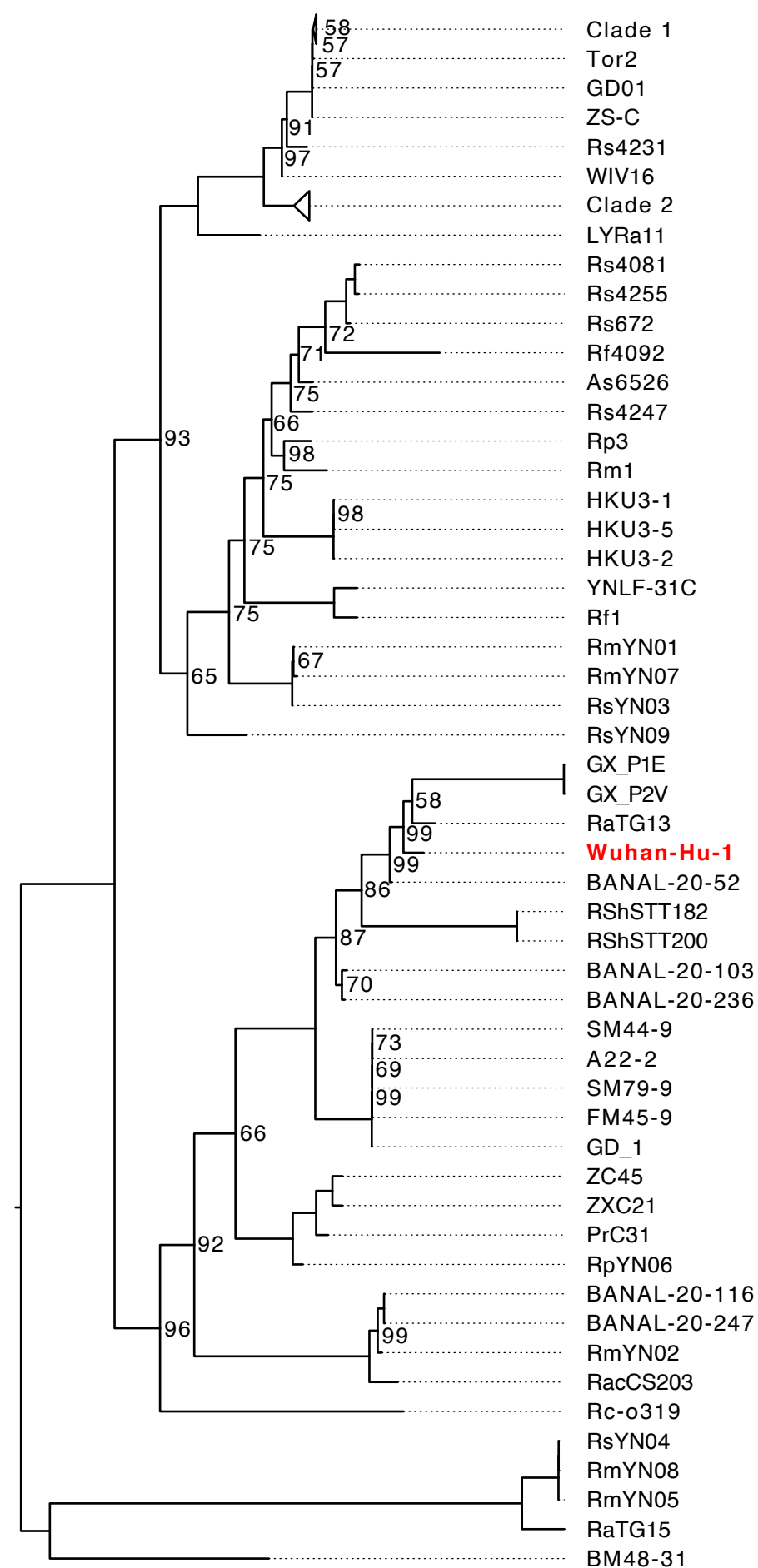

B

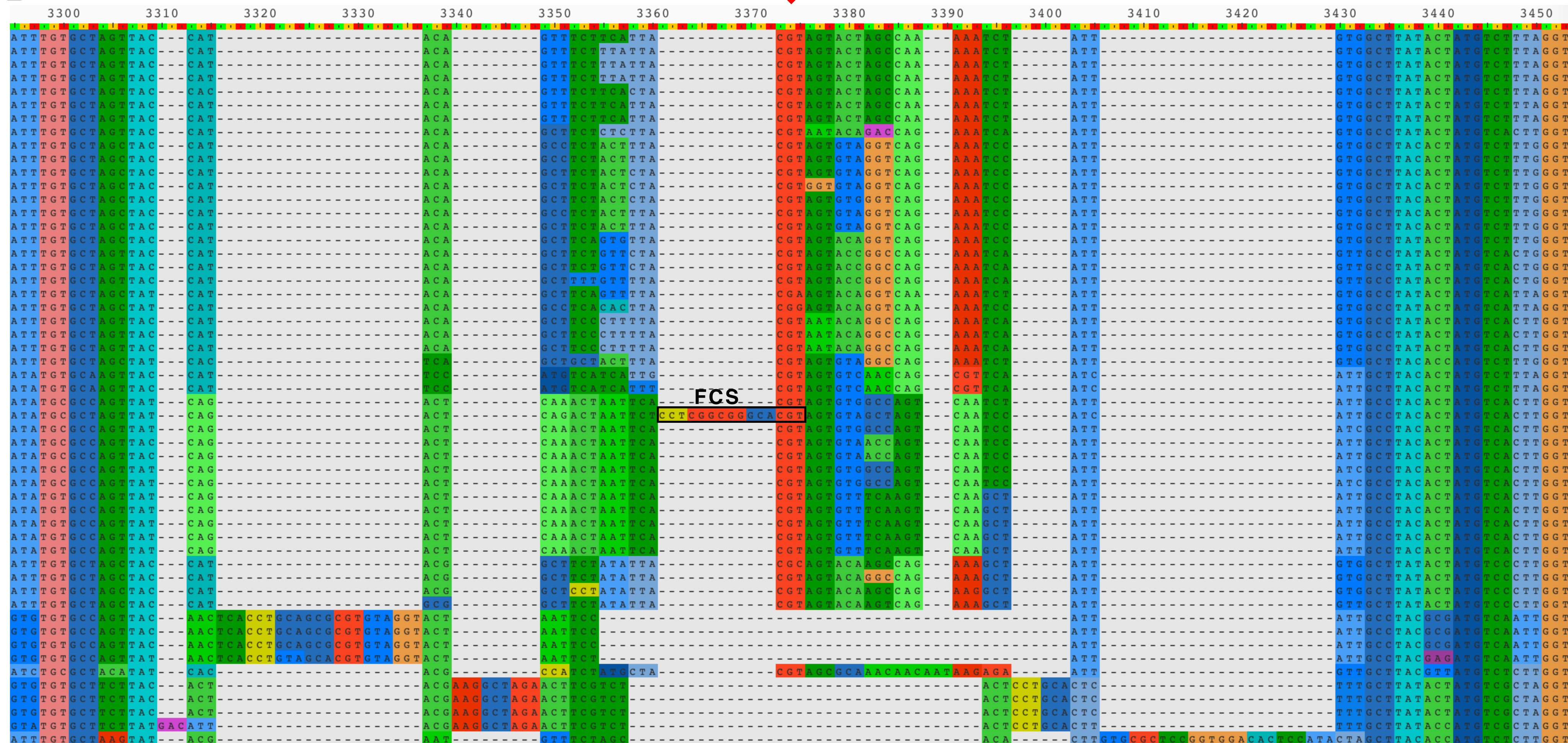

C

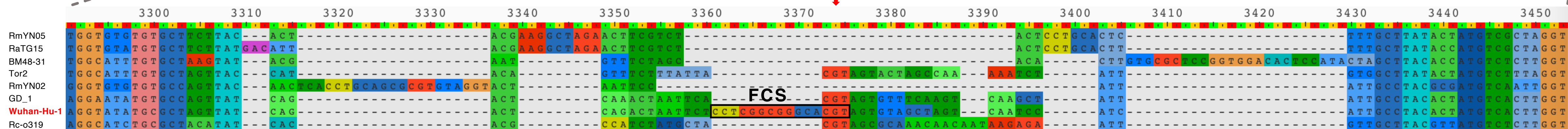

Supplement: msab327_Supplementary_Data [file msab327_supplementary_data.zip › FigureS1_ChanZhan.pdf]
